# Development and content validity testing of a patient-reported outcomes questionnaire for the assessment of hereditary angioedema in observational studies

**DOI:** 10.1186/s12955-015-0292-7

**Published:** 2015-07-01

**Authors:** Nicola Bonner, Linda Abetz-Webb, Lydie Renault, Teresa Caballero, Hilary Longhurst, Marcus Maurer, Sandra Christiansen, Bruce Zuraw, For the Icatibant Outcome Survey (IOS) International Executive Committee and the Hereditary Angioedema Association (HAEA) Medical Advisory Board

**Affiliations:** Adelphi Values, Adelphi Mill, Bollington, Cheshire UK; Shire International GmbH, Zug, Switzerland; University Hospital, La Paz, Hospital La Paz Institute for Health Research (IdiPaz), Biomedical Research Network on Rare Diseases-U754 (CIBERER), Madrid, Spain; Department of Immunology, Barts Health NHS Trust, London, UK; Department of Dermatology and Allergy, Allergie-Centrum-Charité, Charité - Universitätsmedizin Berlin, Berlin, Germany; Medicine Division Allergy/ Immunology and US HAEA Angioedema Center, University of California, San Diego, CA USA; Department of Medicine, Division of Rheumatology, Allergy and Immunology, UC San Diego School of Medicine, San Diego, CA USA; San Diego Veterans Hospital, San Diego, CA USA

**Keywords:** Hereditary angioedema, Patient reported outcome, Questionnaire, Qualitative, Patient, Concept elicitation, Cognitive debriefing, Content validity

## Abstract

**Background:**

Hereditary Angioedema (HAE), a rare genetic disease, manifests as intermittent, painful attacks of angioedema. Attacks vary in frequency and severity and include skin, abdominal and life-threatening laryngeal swellings. This study aimed to develop a patient reported outcome (PRO) tool for the assessment of HAE attacks, including their management and impact on patients’ lives, for use in clinical studies, or by physicians in general practice.

**Methods:**

The results of open-ended face to face concept elicitation interviews with HAE patients in Argentina (*n* = 10) and the US (*n* = 33) were used to develop the first draft questionnaire of the HAE patient reported outcomes questionnaire (HAE PRO). Subsequently, in-depth cognitive debriefing interviews were performed with HAE patients in the UK (*n* = 10), Brazil (*n* = 10), Germany (*n* = 11) and France (*n* = 12). Following input from eight multinational clinical experts further cognitive interviews were conducted in the US (*n* = 12) and Germany (*n* = 12). Patients who experienced abdominal, cutaneous or laryngeal attacks of varying severity levels were included in all rounds of interviews. Across the rounds of interviews patients discussed their HAE attack symptoms, impacts and treatments. Cognitive debriefing interviews explored patient understanding and relevance of questionnaire items. All interviews were conducted face to face following a pre-defined semi-structured interview guide in the patient’s native language.

**Results:**

Patients reported a variety of HAE symptoms, attack triggers, warning signs, attack impacts and treatment options which were used to develop the HAE PRO. The HAE PRO was revised and refined following input from patients and clinical experts. The final 18-item HAE PRO provides an assessment of the HAE attack experience including symptoms, impacts, treatment requirements, healthcare resource use and loss of productivity caused by HAE attacks.

**Conclusions:**

Patient and expert input has contributed to the development of a content valid questionnaire that assesses concepts important to HAE patients globally. HAE patients across cultures consider the HAE PRO a relevant and appropriate assessment of HAE attacks and treatment.

## Background

### Hereditary Angioedema (HAE)

Hereditary angioedema (HAE) due to C1-Inhibitor defects is a rare genetic disease that manifests as unpredictable recurring attacks of painful angioedema (swelling) [1–3]. The disease occurs in two main phenotypic variants: Type I (occurring in approximately 80-85 % of patients) is characterized by a decrease in the formation of C1-INH to about 10-30 % of normal [1]. Type II (approximately 15-20 % of patients) is manifested by production of normal or increased levels of a non-functional C1-INH protein that is antigenically intact [4]. The prevalence of HAE is estimated at 1:50,000 [5].

HAE attacks can occur in various locations of the body but are categorized into three main types: attacks affecting the skin (cutaneous attacks), attacks affecting the gastrointestinal system (abdominal attacks) and attacks affecting the larynx (laryngeal swellings) [4]. Cutaneous attacks are most frequent in occurrence, followed by abdominal attacks, which are known to be extremely painful and laryngeal attacks, which can be fatal if untreated [1, 4, 6]. Attack swellings develop slowly over 36 h and resolve within two to five days [3, 4]. HAE attacks can cause severe discomfort, pain and disability to patients resulting in emotional distress and impacting the patient’s ability to perform their daily activities [7, 8].

A patient reported outcome (PRO) is a measurement report that comes directly from the patient without the input or interpretation of a clinician or any other health professional [9]. PRO measures are important in clinical and registry studies because they provide insight into the patient perspective of the disease experience, and they allow assessment of symptoms and impacts that cannot be measured objectively though the use of traditional biological assessments [10]. This is especially the case in rare disorders, where the disease experience may not be well understood. In HAE particularly, PRO measures are beneficial as the fluctuating nature of HAE attacks can be difficult for physicians to monitor, and for symptoms such as pain, the patient is often the best reporter of the symptoms. Thus in these situations the patient perspective is imperative. When developing a PRO measure, it is essential to first ensure that the measure covers the issues that are relevant to the patients themselves and to ascertain whether the patients understand the questionnaire [9, 11]. Such insights are gathered through qualitative research with patients with the target disease. Without such research, there is a danger that the instrument will be developed to measure concepts that are irrelevant to or misunderstood by patients, which could subsequently yield data that is difficult to interpret.

The identification of bradykinin as the mediator of acute HAE attacks [2] has led to the introduction of new treatments for HAE patients. The approval of new treatments and the need for long term monitoring of safety through registry studies has highlighted the requirement for appropriate HAE assessment tools.

The Icatibant Outcome Survey (IOS) is an international, open-ended patient registry inclusive of all patients who are receiving, or are candidates for, subcutaneous treatment with icatibant, primarily patients with hereditary angioedema [HAE] types I and II [2, 12, 13]. The inclusion of a single instrument assessing all aspects of HAE was proposed as the optimum data collection tool for the study. Thus, the development of a single tool assessing all elements of HAE attacks appropriate for use within the IOS was undertaken.

Published studies have cited the use of PRO measures specific to HAE. Vernon et al. presented the psychometric validation of two PRO measures for use in HAE [14]. However, these two measures are specific to symptom assessment only and therefore, would not be appropriate for the assessment of all elements of a HAE attack including: attack duration, triggers, warning signs, location of attack, symptoms, impacts, resource use and treatment, as is the aim of IOS. Wilson et al. published a study using a web-based survey of HAE patients to determine the economic costs associated with HAE [8]. This PRO, whilst covering a number of relevant concepts, was designed to assess economic burden associated with HAE based on completion at a single timepoint rather than long-term assessments of all elements of HAE attacks. Given that these previously published measures were not considered appropriate for use in the IOS, the development of a new HAE specific PRO tool was undertaken.

The objective of this work was to develop a PRO questionnaire that assesses all aspects of a HAE attack. The specific objective of the first stage of the study was to elicit concepts important and relevant to HAE patients. The objectives of the later stages were to develop a PRO questionnaire assessing all relevant HAE attack concepts and to confirm the face and content validity of the HAE PRO questionnaire.

## Methods

**Fig. 1:**
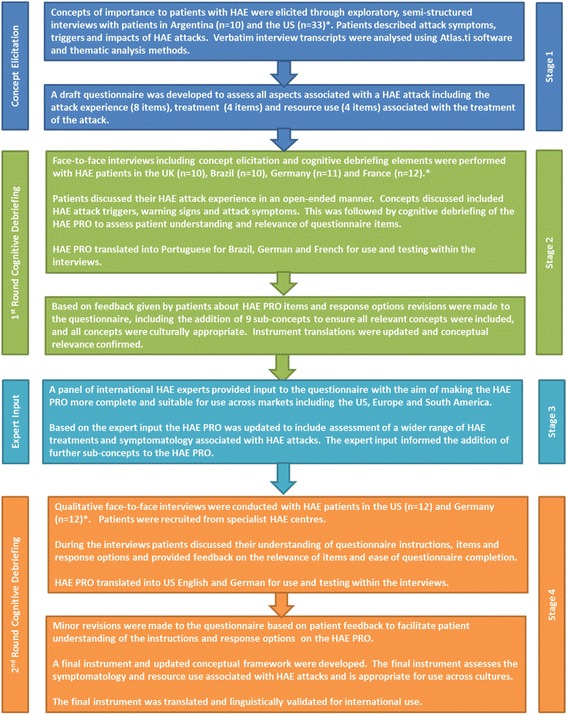
Development of the HAE PRO

The development of the HAE PRO was conducted over four stages involving interviews with HAE patients and consultation with expert HAE clinicians (Fig. 1). At all stages patients were recruited through specialist HAE treatment centers and via patient recruitment agencies and support groups.

### Study participants

For the initial concept elicitation phase of the study, male and female patients aged over 18 years were included if they had a clinician confirmed diagnosis of HAE Type I or II and had experienced a cutaneous, abdominal and/or laryngeal attack within the four weeks prior to screening. The patients were required to be able to complete an interview in their native language (US-English or Spanish for Argentina). Patients were excluded from the study if they fulfilled any of the following criteria: diagnosis of angioedema other than HAE, for example acquired angioedema (AAE); a life-threatening health condition other than HAE; and great difficulty hearing or reading. The same inclusion criterion was used for the second set of concept elicitation interviews and the cognitive debriefing phase of the study.

For the second set of concept elicitation interviews patients were only required to have had a HAE attack within the six months prior to the interview. This criterion was relaxed to aid recruitment of patients with this rare disease.

### Study design

#### Concept elicitation

The first stage of the project involved conduct of qualitative interviews with HAE patients in the US and Argentina. The aim of these interviews was to elicit concepts important to patients to include in the HAE PRO. The open-ended interviews were conducted with 10 patients from one site in Argentina and 22 patients from six sites in the US.

To ensure patients who had experienced a variety of HAE attack types were included in the study the following recruitment quotas were set: Recruitment of up to 10 patients who experienced a cutaneous attack in the four weeks prior to the study, Recruitment of up to 10 patients who experienced an abdominal attack in the four weeks prior to the study and Recruitment of four to eight patients who experienced a laryngeal attack in the four weeks prior to the study. During the interview patients described HAE attack symptoms and how having HAE affects their life, they also discussed their HAE treatment. Patients described HAE attack triggers and how and when they know a HAE attack is coming on (warning signs, prodromal symptoms). Where possible these discussions were conducted in an open-ended manner with the aim to elicit spontaneous responses.

Data from a second set of 11 interviews with HAE patients recruited from two sites in the US provided further evidence of relevant concepts to include in the draft PRO measure. At each study site quotas were set so that at least one patient whose most recent attack was abdominal, cutaneous and laryngeal respectively were targeted for recruitment. Further data pertaining to attack symptoms, impacts, triggers, warning signs and treatments was gathered as part of these interviews.

Using concepts elicited from the two set of interviews a draft questionnaire was developed. The aim of this questionnaire was to assess relevant aspects associated with HAE attacks including the attack experience (8 items), HAE attack treatments (4 items) and resource use (4 items) associated with HAE attacks and its treatment.

### Initial cognitive debriefing interviews

To test the face and content validity of the draft HAE PRO, in-depth, open-ended and cognitive debriefing interviews were performed with HAE patients in the UK (*n* = 10), Brazil (*n* = 10), Germany (*n* = 11) and France (*n* = 12). Interviews were conducted in the local language of each country. In each country at least two patients were recruited for whom their most recent attack was abdominal and cutaneous, respectively, and at least one patient for whom their most recent attack was laryngeal. At this stage, to facilitate recruitment, there was no time limit in which patients had to have had an attack prior to the interview. These cognitive debriefing interviews were divided into two parts. In the first part patients discussed their experience of HAE in an open-ended manner. The second part of the interview involved cognitive debriefing of the draft PRO to assess patient understanding and relevance of questionnaire items, instructions and response options.

Based on patient feedback from this first set of cognitive debriefing interviews revisions were made to the questionnaire items and response options during an international harmonization meeting. Revisions included the addition of nine sub-concepts to ensure inclusion of all concepts relevant to HAE attacks and adaptations to ensure that all concepts were culturally appropriate across countries. The completion of phase two of the research resulted in an instrument that focuses on the symptomatology, treatment and resource use associated with HAE attacks.

### Expert input

Following the first set of cognitive debriefing interviews a panel of international HAE experts (*n* = 8) reviewed the questionnaire with the aim of making the HAE PRO more complete and suitable for use internationally. Following the expert input, the HAE PRO was updated to include assessment of a wider range of HAE treatments (reflective of available treatments in each country) and symptomatology associated with HAE attacks.

### Second round of cognitive debriefing interviews

To confirm the face and content validity and cultural relevance of the revised HAE PRO, further cognitive debriefing interviews were conducted in the US (*n* = 12) and Germany (*n* = 12). During the interviews patients discussed their understanding of the HAE PRO instructions, items and response options and provided feedback on the relevance of items and ease of questionnaire completion. Based on comments from the patients in this final set of interviews and considering feedback from earlier rounds of interviews minor revisions were made to the questionnaire to facilitate patient understanding of the instructions and response options on the HAE PRO. A final instrument and conceptual framework were developed.

### Ethics approval

The study protocols for each phase of the project were submitted to relevant centralized or local ethics review boards, approval was granted for all study phases. All patients completed an informed consent form before undertaking any study activities. Across the individual stages of the study different institutional review boards were involved in the study approvals. These review boards included local site review boards across the US and Argentina, Copernicus Group IRB in the US and the Freiburger Ethik-Komission International in Germany.

### Qualitative analysis of interviews

Across all four project stages interviews were audio recorded and transcribed verbatim. Interview transcripts were translated into English where relevant. Interviews were qualitatively analyzed using Atlas. Ti software [15]. Atlas software allows traditional qualitative analysis of transcripts, but facilitates easier break-down of qualitative data into groups (for example, issues by gender or attack type). Analysis of the concept elicitation interviews focused on identifying HAE concepts important to patients and the language patients’ use to describe those concepts. When analyzing the cognitive debriefing interviews the focus was on confirming patient understanding and interpretation of items, instructions and response options of the HAE PRO. As HAE is a rare condition and patients could be identified from individual descriptive information such as interview location and age all quotes presented in this document specify patient gender only. Across all project stages demographic and clinical data were collected and descriptively analyzed. No other quantitative analysis was conducted as part of this study.

### Translation and linguistic validation

Through all phases of HAE PRO development, consideration was given to ensuring the instrument was appropriate for use across different countries. The instrument has been translated and culturally adapted for use in UK-English, German, French, Italian, Spanish for Argentina, German for Austria and Portuguese for Brazil. At all phases translation has been conducted using two forward, one backwards translation methodology, where two independent forward translations are harmonized and then back-translated to confirm accuracy of the translation. Across all of the translations conceptual equivalence was a key consideration.

## Results

### Stage 1: Initial concept elicitation interviews

The demographic and clinical characteristics of the concept elicitation sample can be found in Table 1.

**Table 1:** Demographic and Clinical Characteristics of the Concept Elicitation Samples (N = 43)

Demographic or clinical characteristic	Initial Concept Elicitation Sample	Second Concept Elicitation Sample
	(*N* = 32)	(*N* = 11)
Demographic Characteristics		
Age		
Mean	39.5	38
Median	39.5	36
Min, Max	20-75	19-54
Gender (*n*) (%)		
Male	9 (28.1)	5 (45.5)
Female	23 (71.9)	6 (54.5)
How would you rate your health in general (*n*) (%)		
Excellent	1 (3.1)	2 (18.2)
Very good	11 (34.3)	4 (36.4)
Good	12 (37.5)	3 (27.3)
Fair	5 (15.6)	1 (9.1)
Poor	3 (9.4)	1 (9.1)
Clinical Characteristics		
Age diagnosed with hereditary angioedema		
Mean	20.3	23.6
Median	20.5	23
Min, Max	1.5-47	7-44
Type of HAE (*n*) (%)		
Type I	28 (87.5)	11 (100)
Type II	4 (12.5)	0 (0)
Type of most recent attack *n* (%)		
Cutaneous	14 (43.8)	5 (45.5)
Abdominal	12 (37.5)	5 (45.5)
Laryngeal	2 (6.3)	0 (0)
Cutaneous and abdominal	3 (9.4)	1 (9)
Cutaneous, abdominal and laryngeal	1 (3.3)	0 (0)
How were you diagnosed with HAE (*n*) (%)		
General Practitioner	4 (3.9)	0 (0)^a^
HAE specialist	14 (43.8)	5 (45.5)
Diagnosed because other family members had the condition	13 (40.6)	4 (36.6)
Other:	1 (3.1)	0 (0)
Allergist	3 (9.4)	0 (0)
Gastroenterologist	1 (3.1)	0 (0)
National Institutes of Health (NIH)	1 (3.1)	0 (0)
Immunologist	1 (3.1)	0 (0)
Pediatrician	1 (3.1)	0 (0)
Research clinic	1 (3.1)	0 (0)
Research physician	0 (0)	1 (9.1)
Physician – internist	0 (0)	1 (9.1)
Rheumatologist	0 (0)	1 (9.1)

^a^n value adds up to more than 11 as one patient reported more than one method of diagnosis

**Fig. 2:**
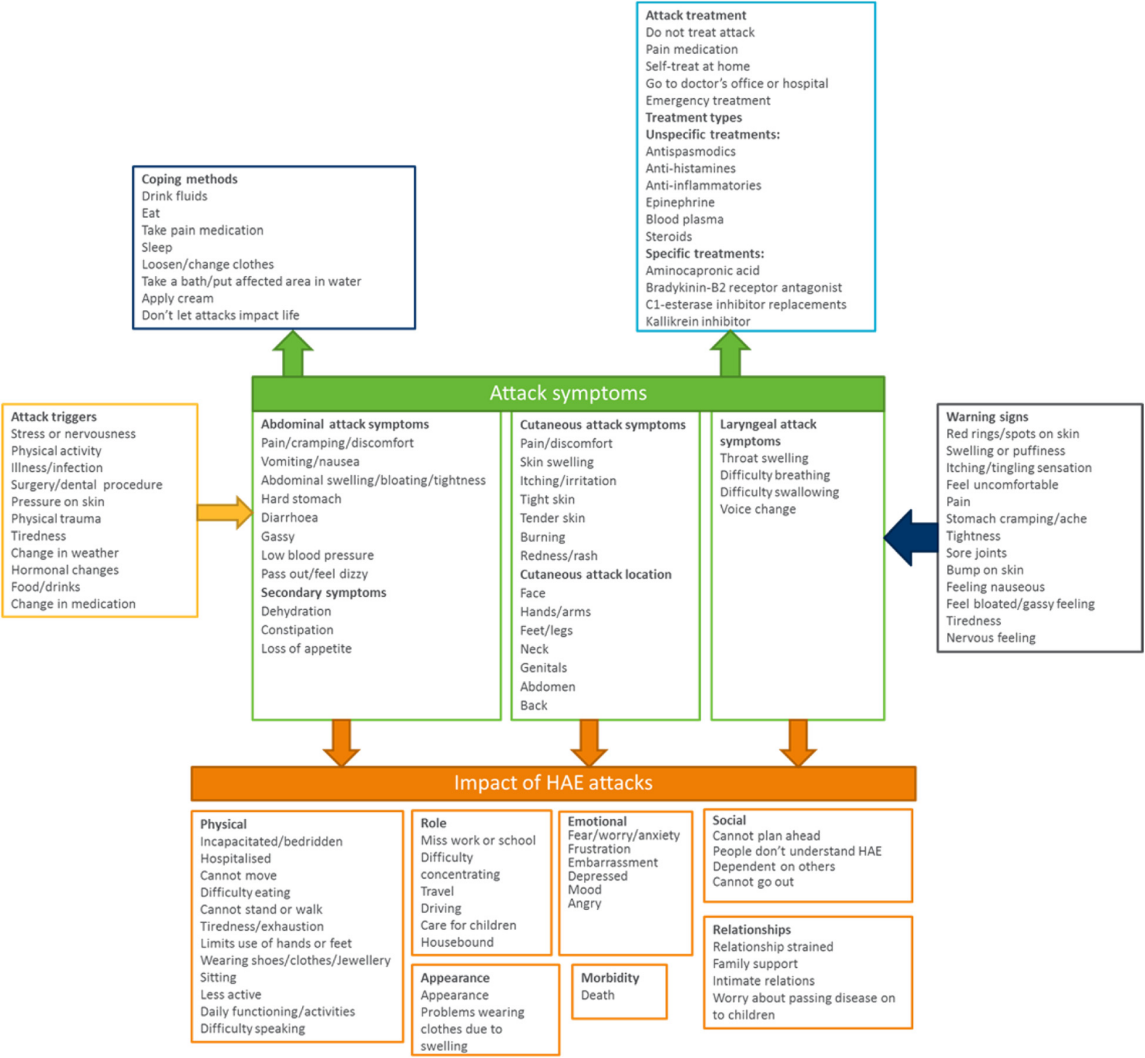
HAE conceptual model based on evidence generated from concept elicitation interviews

A number of concepts emerged from the concept elicitation interviews related to attack triggers, warning signs experienced before attacks, attack symptoms, impacts, attack treatments and coping methods (Fig. 2). Further detail of each concept is presented below.

### Attack triggers

Examples of attack triggers reported by patients included stress, other illnesses, particularly infections, physical activity, surgery, physical trauma and hormonal changes in women (Fig. 2). While some patients were very certain about what triggered their attacks, others stated that attacks sometimes occur with no known trigger.

### Warning signs of HAE attacks

As HAE patients become experienced with their disease they learn to recognize the early warning signs of an attack (Fig. 2). The patients interviewed stated these symptoms appear two to three days before the attack occurs. Examples of warning signs reported include red rings or spots on the skin in the area in which the attack will occur, an itching or tingling sensation in the skin, tight skin, sore joints, a feeling of nausea or a gassy feeling in the stomach and feeling tired. As with triggers, some patients stated that while sometimes they experience recognized warning signs, on other occasions an attack starts without any prior warning.

### Cutaneous HAE attacks

HAE patients described cutaneous attack symptoms including pain or discomfort caused by skin swelling, itching or irritation on the skin and a redness or rash around the area of swelling. The patients also described their skin feeling tight or tender and feeling like the skin is ‘burning’. The reported frequency of cutaneous attacks varied from every few days to once a year. The patients reported that cutaneous attack duration ranged from a few hours to four days; most patients said that the attacks normally last between two and four days. As cutaneous attacks can be relatively mild some patients stated that the duration of the attack can vary based on whether the patient decides to treat it or not. A number of different locations for cutaneous attacks were reported; the most common being the hands, feet, genitals, face (including lips), legs and arms. The patients also reported skin swelling affecting the outer skin of the abdomen which was seen as different to abdominal attacks affecting the gastrointestinal tract.

### Abdominal HAE attacks

The most commonly reported abdominal symptoms were pain, vomiting, swelling, bloating, nausea, diarrhea and cramping. The reported frequency of abdominal attacks was slightly less than cutaneous attacks, ranging from weekly attacks to attacks occurring every month or every two months. Consistent with cutaneous attacks the patients stated that abdominal attacks usually last between two to three days; although some attacks resolve as quickly as 12 h while others last up to a week.

### Laryngeal HAE attacks

Four key laryngeal attack symptoms were consistently described: difficulty breathing, difficulty swallowing, throat swelling and voice change. Laryngeal attacks were reported as less frequent than other HAE attack types. Patients reported only having one or two laryngeal attacks in their life, or only having this type of attack every three or four years. Consistent with other types of HAE attacks, the patients interviewed reported that laryngeal attacks last between a few hours to three or four days.

### Attack severity

While all three types of attack (abdominal, cutaneous and laryngeal) can vary from mild to severe, generally, patients defined attack severity in terms of the type of attack experienced, the symptom’s impact on daily life (i.e., the need to stop doing things) and the need to seek medical care. All laryngeal attacks were considered to be severe; signs and early symptoms of a laryngeal attack led patients to immediately seek medical care since they could potentially experience significant respiratory distress. Laryngeal attacks were viewed as most severe given that they are life threatening. While not as life-threatening as laryngeal attacks, abdominal attacks were considered moderately severe to severe dependent on the level of pain and symptoms experienced. Abdominal attacks were considered more severe than cutaneous attacks. HAE patients reported that skin attack severity varies due to the location of the attack. All cutaneous attacks were considered less painful than abdominal attacks, but severity is dependent on how the attack affects the patient’s ability to function.

### Impact of HAE attacks

As demonstrated in Fig. 2, HAE has a considerable impact on patients’ lives. Patients described a number of impacts that could be grouped into the following sub-concepts: physical impacts, impact on ability to perform usual role, emotional and social impacts, impact on relationships, impact on appearance and morbidity. HAE patients described more severe attacks having a greater impact as they are debilitating, causing the patient to stay at home and miss work and other activities including social plans. Milder attacks, dependent on location, cause less disruption to the patients’ lives. Some patients added that they have learned to live with HAE and try not to let attacks affect their life.

### Attack treatment

The patients stated that they do not always seek treatment if the attack is mild or manageable because they know it will resolve eventually. Other patients stated that they treat themselves at home if they have appropriate treatment available, or just take pain medication to relieve symptoms. For more severe attacks, patients seek treatment from their doctor, and particularly in the case of laryngeal attacks seek emergency treatment. Some patients described experiences where they had tried to seek treatment for an attack but were given inappropriate treatment because doctors were unfamiliar with HAE. In some cases treating doctors even tried to do surgery to treat abdominal attacks, actually exacerbating the attack. The patients described specific treatments they had taken for their HAE as presented in Fig. 2.

### Conceptual model

Data generated from the concept elicitation interviews was used to develop a conceptual model for HAE and acute attacks (Fig. 2). The conceptual model provides a summary of HAE attack symptoms, impact domains, attack triggers and warning signs, coping methods employed by patients and HAE treatments. The conceptual model demonstrates the complexity of HAE and illustrates the need to consider all aspects of the attack and not just symptoms.

### Stage 2: First round of cognitive debriefing interviews

The demographic and clinical characteristics of the initial cognitive debriefing sample can be found in Table 2.

**Table 2:** Demographic and Clinical Characteristics of the Initial Cognitive Debriefing Sample (N = 43)

	UK (*n* = 10)	Brazil (*n* = 10)	Germany (*n* = 11)	France (*n* = 12)	Total (*n* = 43)
Demographic Characteristics					
Age of patient					
Mean	46	38	38	36	39
Min - Max	20-66	24-66	18-65	20-60	18 - 66
Gender *n* (%)					
Male	2 (20)	2 (20)	5 (45)	6 (50)	15 (35)
Female	8 (80)	8 (80)	6 (55)	6 (50)	28 (65)
Highest education level^a^ *n* (%)					
General Certificate of Secondary Education (GCSE) or less	6 (60)	0	1 (9)	0	7 (16)
Advanced (A) Levels	2 (20)	6 (60)	1 (9)	1 (8)	10 (23)
Vocational qualification or Apprenticeship	0	0	5 (45)	1 (8)	6 (14)
University or College degree	2 (20)	2 (20)	4 (36)	4 (33)	12 (28)
Post-graduate degree or qualification	0	2 (20)	0	6 (50)	8 (19)
Clinical Characteristics					
Type of Hereditary Angioedema *n* (%)					
Type I	N/A^b^	9 (90)	9 (82)	9 (75)	27 (82)
Type II	N/A	1 (10)	2 (18)	1 (8)	4 (12)
Missing data	N/A	0	0	2 (17)	2 (6)
Type of Hereditary Angioedema attack(s) experienced in the past six months *n* (%)					
Cutaneous edema (skin swelling)	9 (90)	8 (80)	10 (91)	10 (83)	37 (86)
Abdominal edema (internal swelling)	7 (70)	6 (60)	9 (82)	9 (75)	31 (72)
Laryngeal edema (throat swelling)	5 (50)	2 (20)	3 (27)	2 (17)	12 (28)
Type of Hereditary Angioedema attack(s) experienced most recently *n* (%)					
Cutaneous edema (skin swelling)	5 (50)	6 (60)	7 (64)	8 (67)	26 (60)
Abdominal edema (internal swelling)	7 (70)	3 (30)	6 (55)	7 (58)	23 (53)
Laryngeal edema (throat swelling)	2 (20)	1 (10)	0	0	3 (7)
Days since last attack					
Mean	44	33	17	75	43
Min - Max	0 - 172	4 - 133	0 - 84	9 - 204	0 - 204
Hereditary Angioedema diagnosis by *n* (%)					
General Practitioner (GP)	3 (30)	0	1 (9)	0	4 (9)
Hereditary Angioedema Specialist	3 (30)	5 (50)	5 (45)	3 (25)	16 (37)
Diagnosed because other family members had the condition	2 (20)^c^	1 (10)	4 (36)	5 (42)	12 (28)
Diagnosis due to other tests/problems	2 (20)	0	1 (9)	0	3 (7)
Dermatologist	1 (10)	0	0	1 (8)	2 (5)
Pediatrician	0	0	0	1 (8)	1 (2)
Allergist	0	2 (20)	0	1 (8)	3 (7)
Emergency Physician	0	1 (10)	0	1 (8)	2 (5)
Neurologist	0	1 (10)	0	0	1 (2)

^a^Educational levels are provided as described for the English interviews. The levels were translated and made culturally equivalent for each country

^b^Data for Type of HAE not available for UK patients as this was a clinician reported characteristic, no clinician reported data was collected in the UK

^c^One patient chose diagnosed by GP and diagnosed because other family member had the condition

Table 3 presents the symptoms assessed by the HAE PRO that were spontaneously reported by patients and the additional symptoms reported that were not specifically included in the first version of the HAE PRO. All symptoms measured by the HAE PRO questionnaire were mentioned by at least three patients. The most commonly mentioned symptoms were assessed by the HAE PRO, including abdominal pain (*n* = 30), skin swelling (*n* = 28), vomiting (*n* = 15), diarrhea (*n* = 13), nausea (*n* = 11) and skin pain (*n* = 11). A number of additional symptoms were spontaneously reported by patients. Of these symptoms, it was agreed that difficulty breathing (*n* = 5) and constipation (*n* = 3) should be included in the HAE PRO due to perceived importance to patients and clinical relevance. While a number of other symptoms were reported they were considered similar to other symptoms already included in the questionnaire, or distal, secondary symptoms that were not necessary to add.

**Table 3:** Assessment of coverage of HAE symptoms by the HAE PRO

Symptoms assessed by HAE PRO and mentioned by patients	Additional symptoms mentioned by patients (n = 43)
Abdominal pain (n = 30)	Stomach/bowel swelling (n = 13)
Skin swelling (n = 28)	Spasms/cramping (n = 8)
Vomiting (n = 15)	Tiredness (n = 8)
Nausea (n = 11)	Stomach ache (n = 6)
Diarrhoea (n = 13)	Difficulty breathing (n = 5)
Skin pain (n = 11)	Sore skin (n = 4)
Erythema (skin redness) (n = 8)	Constipation (n = 3)
Skin irritation (n = 5)	Tingling (n = 3)
Voice change (n = 4)	Tight skin (n = 3)
Difficulty swallowing (n = 3)	Red rings (n = 2)
	Itching (n = 2)
	Burning (n = 2)
	Discomfort (n = 2)
	Passing out/drop in blood pressure (n = 2)
	Tight esophagus (n = 2)
	Throat pain (n = 2)

Table 4 presents the HAE attack triggers assessed by the HAE PRO that were spontaneously reported during the interview, and the additional triggers reported by patients that were not specifically included in the first version of the HAE PRO. All triggers included in the HAE PRO questionnaire were confirmed by at least one patient. From the additional triggers mentioned by patients, tiredness (*n* = 8) was added to the revised questionnaire.

**Table 4:** Assessment of coverage of HAE attack triggers by the HAE PRO

Triggers assessed by HAE PRO and mentioned by patients	Additional triggers mentioned by patients (n = 43)
Emotional distress (n = 18)	Tiredness (n = 8)
Physical trauma (n = 15)	Temperature (low, high, sudden change) (n = 4)
Hormones (n = 12)	Shoes (n = 3)
Pressure on the skin (n = 11)	Dental treatment (n = 2)
Illness/ infection (n = 10)	Insect bite (n = 2)
None (n = 7)	Sunlight (n = 1)
Stress (n = 6)	Sport (n = 1)
Sitting or standing (n = 5)	Repeated movement (n = 1)
Food or drink (n = 4)	Smoke (n = 1)
Chemicals (n = 2)	
Medication (n = 1)	

Coverage of HAE warning signs included in the draft HAE PRO and reported by patients spontaneously can be found in Table 5. All warning signs presented on the HAE PRO were supported by the patients. From the additional attack warning signs mentioned, red rings on the skin (*n* = 3) was added to the questionnaire as this was considered by patients to be an important warning sign of an attack.

**Table 5:** Assessment of coverage of HAE attack warning signs by the HAE PRO

Warning signs assessed by HAE PRO and mentioned by patients	Additional warning signs mentioned by patients (n = 43)
Skin redness (n = 13)	Pain (n = 4)
Irritability (n = 11)	Itchiness (n = 4)
Nausea (n = 8)	Burning (n = 4)
Skin sensation (n = 7)	None (n = 4)
Tiredness (n = 7)	Feeling of butterflies (n = 3)
Skin Tightness (n = 6)	Swelling (n = 3)
Aggressiveness (n = 3)	Red rings on the skin (n = 3)
Sensitivity to noise (n = 3)	Stress (n = 2)
Hunger (n = 2)	Headaches (n = 2)
Prickling (n = 1)	Thirst (n = 2)
	Difficulty breathing (n = 2)
	Numbness (n = 2)

Cognitive debriefing of the HAE PRO indicated that on the whole, the instrument was well understood by HAE patients. The patients interpreted the items and response options consistently and found that the HAE PRO accurately captured their experiences of HAE attacks and the treatment they receive. Although the HAE PRO was well received, patients made recommendations to further improve the face and content validity of the HAE PRO. These changes were implemented to further strengthen the face and content validity of the instrument.

### Stage 3: Expert input

Between the first and second set of cognitive debriefing interviews a panel of international HAE experts provided input to the questionnaire. Based on this input the HAE PRO was updated to include assessment of a wider range of HAE treatments and symptomatology, specifically a number of items assessing preventative HAE treatments were added to the instrument.

### Stage 4: Second round of cognitive debriefing interviews

Details of the demographic and clinical characteristics of the second cognitive debriefing sample can be found in Table 6.

**Table 6:** Demographic and Clinical Characteristics of the Second Cognitive Debriefing Sample (N = 24)

	Germany (n = 12)	US (n = 12)	Total (n = 24)
Demographic Characteristics			
Age of patient			
Mean	40.6	42.5	41.5
Min - Max	23-61	21-60	21-61
Gender *n* (%)			
Male	3 (25)	6 (50)	9 (37.5)
Female	9 (75)	6 (50)	15 (62.5)
Highest education level *n* (%)			
Some high school	1 (8.3)	0	1 (2.4)
High school diploma or GED	2 (16.7)	3 (25)	5 (20.8)
Some years of college	3 (25)	3 (25)	6 (25)
Vocational qualification or Apprenticeship	5 (41.7)	1 (8.3)	6 (25)
University or College degree	1 (8.3)	4 (33.3)	5 (20.8)
Post-graduate degree or qualification	0	1 (8.3)	1 (4.2)
Clinical Characteristics			
Type of Hereditary Angioedema *n* (%)			
Type I	11 (91.7)	12 (100)	23 (95.8)
Type II	1 (8.3)	0	1 (4.2)
Type of Hereditary Angioedema attack(s) experienced in the past six months^a^ *n* (%)			
Cutaneous edema (skin swelling)	10 (83.3)	11 (91.6)	21 (87.5)
Abdominal edema (internal swelling)	8 (66.7)	7 (29.2)	15 (62.5)
Laryngeal edema (throat swelling)	2 (16.6)	2 (16.6)	4 (16.7)
Type of Hereditary Angioedema attack(s) experienced most recently^a^ *n* (%)			
Cutaneous edema (skin swelling)	9 (75)	8 (66.7)	17 (70.8)
Abdominal edema (internal swelling)	5 (41.6)	5 (41.6)	10 (41.6)
Laryngeal edema (throat swelling)	0 (0)	0 (0)	0 (0)
Days since last attack			
Mean	784	64.25	424
Min – Max	1-8604	3-405	1-8604
Hereditary Angioedema diagnosis by *n* (%)			
General Practitioner (GP)	1 (8.3)	2 (16.7)	13 (12.5)
Hereditary Angioedema Specialist	7 (58.3)	5 (41.7)	12 (50)
Diagnosed because other family members had the condition	4 (33.3)	4 (33.3)	8 (33.3)
Other	0 (0)	1 (8.3)	1 (4.2)

^a^Patients could select more than one type of attack

Consistent with the first round of cognitive debriefing interviews, overall, the HAE PRO was relevant to HAE patients and deemed appropriate for their attack experience. While there were some problems knowing how to complete some of the items, the majority of these were likely due to patients completing the questionnaire out of context of a study, not in the intended mode of administration and when they had not necessarily had an attack in the days prior to the interview. Although the majority of patients were happy with the HAE PRO there were some items, instructions and response options that caused patients a considerable amount of difficulty therefore some revisions were made to the instrument to enhance the understandability and relevance of the instructions, items and response options of the instrument.

### Final HAE PRO

**Fig. 3:**
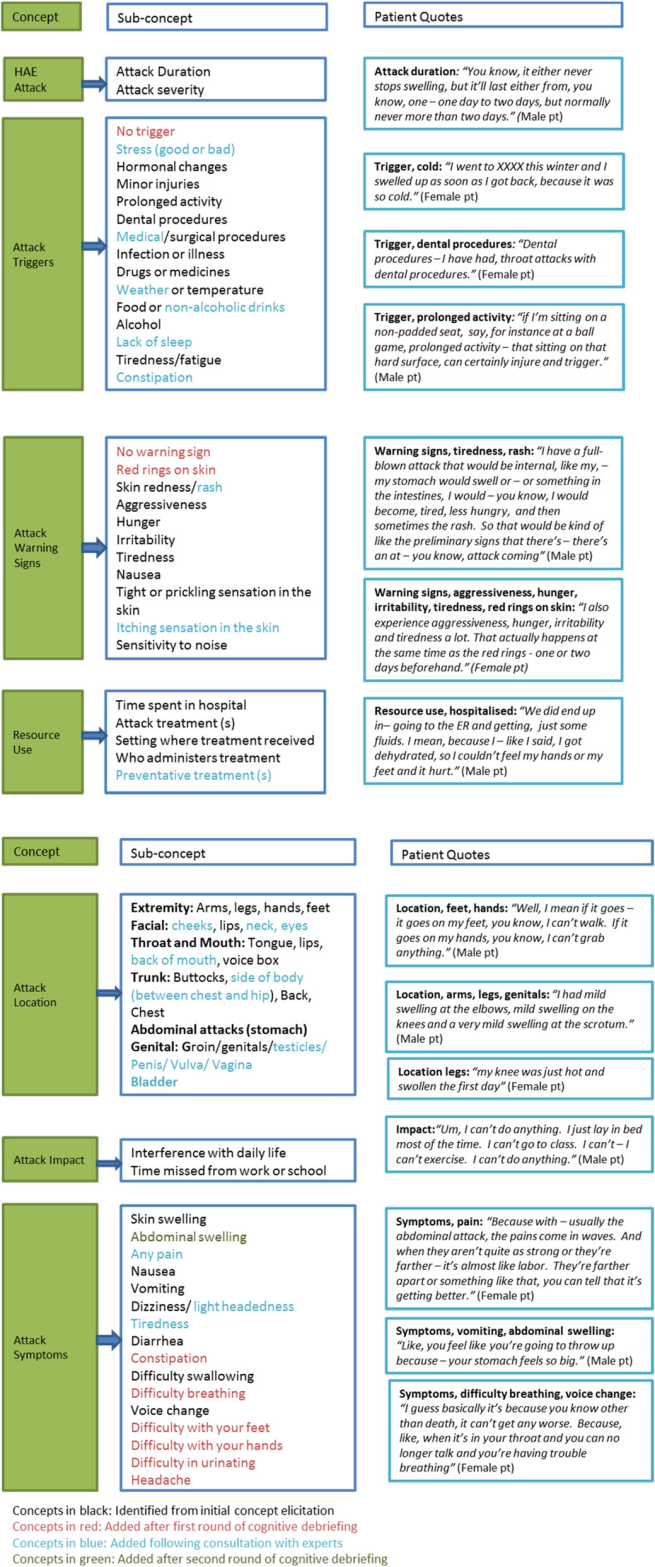
HAE PRO conceptual framework

Evidence from the second round of cognitive debriefing interviews was considered alongside patient input from the previous phases of the research and a final instrument was created. Figure 3 presents the conceptual framework for the final instrument with patient quotes confirming the relevance of the HAE PRO. The concepts in the model are color coded to demonstrate the evolution of the instrument over time.

## Discussion

Evidence generated from the concept elicitation phase demonstrated the complex, multi-faceted nature of HAE. Not only do patients experience different symptoms depending on attack type, their lives are considerably impacted by the disease. Patients have to manage living with an unpredictable genetic condition whilst coping with a lack of awareness of the disease by clinicians and members of the public.

Given the complex nature of HAE it was clear that multiple HAE concepts need to be assessed to achieve a holistic assessment of HAE attacks and their treatment. As there were no existing measures assessing a range of factors specific to HAE including triggers, warning signs, symptoms, impact and resource use the development of an instrument assessing a range of HAE concepts was undertaken.

To ensure all concepts important to patients were captured concept elicitation interviews were conducted with HAE patients in two countries. The concept elicitation interviews highlighted that the patient experience of HAE extends beyond HAE attacks alone. Evidence from the concept elicitation interviews allowed a comprehensive conceptual model to be developed that captures HAE attacks in their entirety, including concepts such as symptoms, impacts, treatment and resource use associated with HAE.

Face and content validity of the instrument was tested through cognitive debriefing interviews with HAE patients in four countries. Analysis of the concept coverage of the HAE PRO questionnaire confirmed that all concepts included in the measure are relevant to HAE patients across different attack types. Although, the HAE patients highlighted additional concepts above those already captured by the instrument, most of the additional symptoms, triggers or warning signs described by patients were seen as similar to those already included, or secondary symptoms captured by existing items on the questionnaire. However, where it was considered clinically relevant or there was substantial patient support additional concepts were added. Where patients experienced triggers, warning signs or symptoms not directly assessed they were satisfied that these could be reported under ‘other’. This evidence from the open-ended aspects of the initial cognitive debriefing interviews supports the content validity of the HAE PRO.

While the questionnaire was generally well understood and interpreted correctly and consistently, there were some aspects of the items and response options that caused confusion for the patients during the first round of cognitive debriefing interviews. As a follow up to the cognitive debriefing study an international harmonization meeting was held involving representatives from each language in which the cognitive debriefing study was performed. In this meeting each item was discussed and patient specific feedback was considered. Revisions to all relevant aspects of the questionnaire were made to increase understanding and relevance of all of concepts and items included in the questionnaire, without adding significant respondent burden. These revisions further support the face and content validity and global relevance of the questionnaire.

The World Allergy Organization (WAO) Guideline for the Management of HAE [16] and the Hereditary Angioedema International Working Group (HAWK) consensus report provide evidence-based recommendations regarding management approaches for HAE patients. These documents, published in 2012, were important considerations to ensure that the questionnaire addresses the key issues for HAE patients and physicians [17]. As these documents were published after the initial development of the HAE PRO, to confirm the clinical relevance of the instrument, expert HAE clinicians reviewed the HAE PRO against the WAO Guideline for the Management of HAE and the HAWK consensus report.[16, 17] The clinical experts further expanded the conceptual coverage of the HAE PRO to ensure complete assessment of the HAE-related issues that may not have been initially raised by the patients themselves, but that were deemed to be clinically relevant to HAE. A key addition was the inclusion of items assessing preventative HAE treatment as preventative treatment options have changed since the initial interviews. These items were considered important to accurately reflect resource use associated with HAE and its treatment.

A second set of cognitive debriefing interviews confirmed the relevance of revisions made to the HAE PRO following the clinician input. Evidence from these interviews confirmed that the HAE PRO is a relevant, well understood measure of HAE attack concepts. Several minor edits implemented based on the interview findings will further strengthen patient comprehension and reduce the likelihood of confusion when completing the questionnaire in a real world setting.

The face and content validity of the HAE PRO has been confirmed across four stages of development using input from 110 HAE patients from six countries. Given the global base of the sample the authors are confident that the conceptual model used as the basis for development of the instrument is representative of HAE patients’ experiences and the findings of the study are generalizable across cultures. The culturally diverse sample that provided input, particularly in the cognitive debriefing stage of the HAE PRO development supports the use of the HAE PRO in a multi-national study. Throughout the development process consideration was given to ensuring items and response options were reflective of the attack experience across countries and not simply specific to one culture or healthcare system. The HAE PRO is considered a content valid and appropriate tool for the long term assessment of HAE attack symptoms and resource use in a real world context.

Since development of the HAE PRO, two questionnaires assessing health-related quality of life in HAE patients have been developed, the HAE-QoL and the AE-QoL [18, 19]. However, while both of these instruments are well developed and specific to HAE (HAE-QoL) or recurrent angioedema including HAE (AE-QoL), neither assesses all of the concepts identified in the concept elicitation phase of this work and their aims are different. The HAE-QoL, developed by Prior et al., assesses very specific impacts of HAE on quality of life, but there is no assessment of concepts specifically related to HAE attacks including: triggers, warning signs, symptoms, attack characteristics and treatments [19]. The AE-QoL, developed by Weller et al., is a very well developed tool that focuses more on the impacts of angioedema and how having the condition affects the patients, rather than assessing specific HAE attack characteristics [18]. While these instruments both have their interest to assess how HAE affects patients, they are not considered for independent use in the assessment of HAE attacks as a whole. As the aim of this project was to develop a questionnaire that could be used to assess all aspects of HAE in a real world context the HAE PRO is considered the most appropriate instrument to take forward to the proposed registry study.

While this was a robust, well-designed study certain limitations in the study design and ability to make distinct conclusions should be recognized. While the HAE PRO development process involved patients from across a number of different countries, the country representation and patient involvement differed at each of the development stages. This was partly intentional to ensure representative input from a range of different cultures and healthcare settings was achieved at the different phases of HAE PRO development. However, this was also a factor of the rare nature of HAE and the ability to recruit sufficient numbers of independent patients in each country for each phase of the instrument development. While there were variations in the involvement of patients from each of the countries represented at the different phases of the research, the authors feel this allowed a greater cultural representation in the HAE PRO development process and findings indicated that concepts of importance to patients were consistent across the countries. However, it is recognized that this variation in country representation limits the distinct conclusions that can be made about relevance of concepts to each individual country of interest.

The face and content validity of the HAE PRO has been confirmed through this extensive qualitative research process. However, the measurement properties of the HAE PRO have not been explored. Future additional research could be conducted to confirm the measurement properties of the HAE PRO. Furthermore, currently the HAE PRO has been developed and culturally adapted for use across seven different languages, further translation and linguistic validation could be conducted to expand the HAE PRO for use in additional language versions for additional country settings.

## Conclusion

Input from HAE patients and expert clinicians has contributed to the development of a strong, content-valid questionnaire that assesses concepts important to HAE patients globally. HAE patients across cultures consider the HAE PRO a relevant and appropriate assessment of concepts associated with HAE attacks and preventative treatments. The HAE PRO can be considered a content-valid and appropriate tool for the long-term assessment of HAE attack symptoms and resource use in a real-world context.

For further information about the HAE PRO please contact Shire (haepro@shire.com).

## Funding and acknowledgements

The work was funded and supported by Shire, Zug Switzerland. Adelphi Values were employed by Shire as expert consultants to conduct the patient interviews, analyse the data, develop the questionnaire and develop the manuscript. Editorial support was provided by Adelphi Values funded by Shire, Zug, Switzerland.

The Icatibant Outcome Survey (IOS) Executive Committee and the Hereditary Angioedema Association (HAEA) Medical Advisory Board reviewed the draft HAE PRO and provided clinical input.

Teresa Caballero, Hilary Longhurst, Marcus Maurer, Werner Aberer, Andrea Zanichelli, Laurence Bouillet are members of the Icatibant Outcome Survey Executive Committee, Shire.

Sandra Christiansen and Bruce Zuraw are members of the Hereditary Angioedema Association (HAEA) Medical Advisory Board.

## Abbreviations

AAE: Acquired Angioedema
HAE: Hereditary Angioedema
HAW: Hereditary Angioedema International Working Group
IOS: Icatibant Outcome Survey
PRO: Patient Reported Outcome
WAO: World Allergy Organization
